# Effect of Helium Plasma Exposure on Wettability and Shear Bond Strength between the Zirconia Core and Feldspathic Veneering Ceramic: An In Vitro Study

**DOI:** 10.1155/2022/6831864

**Published:** 2022-06-24

**Authors:** Niyousha Rafeie, Tabassom Hooshmand, Parham Pedram

**Affiliations:** ^1^Department of Dental Biomaterials, School of Dentistry, Tehran University of Medical Sciences, Tehran, Iran; ^2^Department of Dental Biomaterials, School of Dentistry/Research Center for Science and Technology in Medicine, Tehran University of Medical Sciences, Tehran, Iran

## Abstract

**Introduction:**

The present study aimed to evaluate the effect of helium plasma treatment on the wettability of zirconia surface and on the shear bond strength between the dental zirconia core and feldspathic veneering ceramic.

**Methods:**

128 zirconia specimens were prepared, polished, and then divided into four groups: control, Zr, FC, and Zr/FC. In Zr and Zr/FC groups, the zirconia blocks were treated by helium plasma for 60 s. In FC and Zr/FC, the feldspathic ceramic powder received 60 s of plasma treatment. Then, the feldspathic powder was applied on the zirconia blocks. Half of the specimens in each group were sintered in a tube furnace, and the contact angle between the zirconia core and feldspathic ceramic was measured at different time intervals. The other half were sintered in a ceramic furnace and then subjected to thermocycling. The shear bond strength was measured using a universal testing machine. The failure mode was assessed using a stereomicroscope. Data were analyzed by one-way ANOVA test, and the statistical significance was considered less than 0.05.

**Results:**

There was no significant difference in the mean contact angle and the shear bond strength values of the experimental groups (*P* > 0.05). The mean contact angle decreased significantly in all groups over time (*P* < 0.001). The modes of failure were predominantly mixed in all groups.

**Conclusion:**

The helium plasma applied on either dental zirconia core or feldspathic ceramic powder could not improve the zirconia surface wettability and the shear bond strength between the two ceramics.

## 1. Introduction

In recent years, the definitions of esthetic have changed among patients and clinicians [1]. Therefore, metal-ceramic restorations are being replaced by more esthetic restorations such as all-ceramic crowns to restore damaged teeth structures [2, 3]. In addition, some clinical disadvantages have been attributed to metal-ceramic restorations, for example, inducing an allergic or toxic reaction in adjacent tissues [4], marginal grey discoloration in adjacent gingiva due to the metal component [5], loss of vitality of abutment teeth, and more cases of abutment fractures [6]. In contrast, all-ceramic restorations are biocompatible and do not induce allergic or toxic reactions and gingival discoloration as opposed to metal-ceramic restorations [7]. Generally, in all-ceramic restorations, a high-strength ceramic such as zirconia is used as a substructure/core, and a layer of esthetic ceramic such as feldspathic ceramic is applied on the core to cover the zirconia opacity. However, chipping and delamination of veneering ceramic are a major challenge in zirconia-veneer restorations, which threaten their longevity [8,9]. Therefore, it is crucial to enhance the bond between the zirconia core and veneering ceramic in order to improve the long-term clinical success [10].

Various surface modification techniques have been recommended to improve the bond between the zirconia and veneering ceramic mechanically and chemically such as sandblasting with different particle sizes and different oxides [11–14], mechanical grinding, heat treatment [15], laser [16, 17], and application of nonthermal plasma [18]. However, some of these techniques not only do not improve the bond strength, but also affect the zirconia qualities adversely. For instance, sandblasting decreases the zirconia strength by inducing tetragonal to monoclinic phase transformation [19]. Other surface treatment techniques such as liner application, silica coating, and laser treatment have shown disputable effects on the bond strength [17, 20–23]. These controversies mark a need for a more reliable surface treatment method to enhance the interfacial bond between zirconia and feldspathic veneer without affecting the qualities of zirconia.

Recently, plasma technology has been introduced to dental practice, and its potential for improving bond strength has captured researchers' attention; at first, plasma treatment was used to improve the bond strength between the zirconia and resin cement interface [24–27]. Lately, plasma exposure has been proposed as a method to improve the bond strength between the zirconia core and veneering ceramic. According to recent studies, the application of atmospheric pressure nonthermal argon plasma has enhanced the interfacial bond between the zirconia-veneering ceramic [18, 28, 29]. This method has many advantages over other surface treatment techniques, such as its easy application [30]. Moreover, the plasma does not affect the zirconia surface negatively as opposed to sandblasting [28]. Nonthermal argon plasma can significantly reduce the organic matter and generate reactive species on the surface [31, 32]. According to Ji et al. [28], 10 min of argon plasma exposure could significantly increase the bond strength of veneering ceramic to zirconia core. In another study conducted by Liu et al. [29], both methane (CH_4_) and argon plasma could significantly improve the zirconia-veneering ceramic bond strength.

To the best of our knowledge, no study has investigated the effect of helium plasma on this bond. Besides, it has been suggested that the helium plasma jet is more efficient compared to argon, since the difference between the metastable state and ionization energy for helium plasma is 3.9 eV which is less than argon ionization energy (4.3 eV) [33]. In other words, higher metastable energy of helium compared to argon plasma (20.06 and 11.60 eV, respectively) enables helium plasma to generate more reactive species compared to argon. The superior performance of helium plasma regarding improving the hydrophilic characteristics of the surface has been reported by Wang et al. [34]. According to their results, helium plasma was more effective in improving the hydrophilic properties of polymethyl methacrylate (PPMA) and also produced more active species compared to the argon plasma.

Therefore, the hypothesis of this study was that the helium plasma might be more efficient in improving the hydrophilic properties of the zirconia surface, thus leading to a stronger bond between the zirconia and veneering ceramic. In addition, due to the higher efficacy of helium plasma jet device compared to argon and the lack of adequate information regarding the effect of helium plasma on the bond strength, the present study aimed to evaluate the effect of nonthermal helium plasma treatment on the wettability of zirconia surface and the shear bond strength between the zirconia core and feldspathic veneering ceramic.

## 2. Materials and Methods

### 2.1. Sample Size

The minimum sample size was calculated to be 14 in each experimental group according to a similar study by He et al. [35], using one-way ANOVA feature of PASS 11 software (NCSS, LLC, Kaysville, Utah, USA), considering alpha = 0.05, beta = 0.2, and mean standard deviation of shear bond strength equal to 6.27. (The selected sample size in this study was 16.)

### 2.2. Specimen Preparation

**Figure 1:**
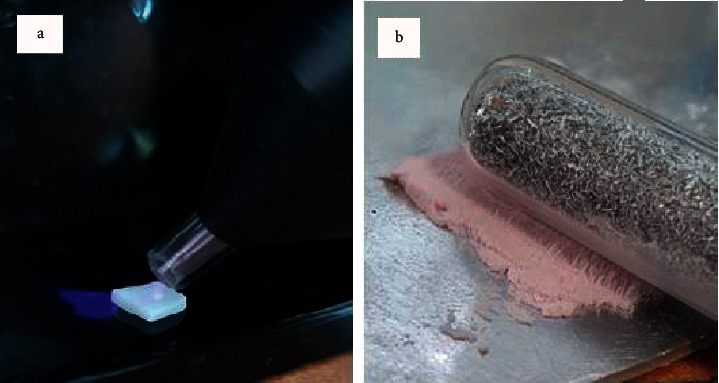
Application of nonthermal helium plasma on (a) zirconia core and (b) veneering ceramic powder.

From two pre-sintered blocks of zirconia-based ceramic (Prettau, Zirkonzahn, Italy, lot number ZB6138E), one hundred and twenty-eight zirconia specimens were cut in dimensions of 10 × 10 × 2 mm by a low speed saw (PELCO Precision, Ted Pella, Inc., Stockholm, Sweden) and then polished with 2500 grit silicon carbide sandpaper [36]. The zirconia specimens were sintered for 1 h at 1600°C according to the manufacturer's instructions. Then, the specimens were cleaned ultrasonically for 15 min in ethanol [29] and divided randomly into four experimental groups (*n* = 32) as follows:

- Control group: zirconia + feldspathic ceramic, both without surface treatment.
- Zr group: zirconia with plasma surface treatment + feldspathic ceramic without surface treatment (Figure 1(a)). Zirconia blocks were treated with nonthermal helium plasma surface treatment at a distance of 10 mm for 60 s [37].
- FC group: zirconia without surface treatment + feldspathic ceramic with plasma surface treatment. Feldspathic ceramic powder was treated by nonthermal helium plasma for 60 s and the nozzle was in direct contact with the ceramic powder (Figure 1(b)).
- Zr/FC group: zirconia + feldspathic ceramic, both with plasma surface treatment.

A nonthermal helium plasma device (Medaion, Nikfannavaran Plasma Co., Tehran, Iran) with flow rate of 10 L/min was used. The frequency and power of the device were 50 kHz and 10 W, respectively [28].

### 2.3. Contact Angle Measurement

**Figure 2:**
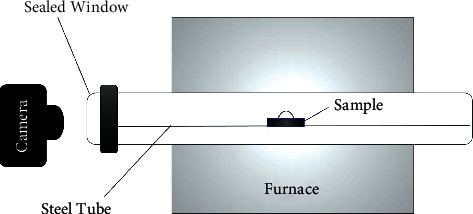
Schematic image showing how the images were obtained when zirconia-veneering ceramic assemblies were in the furnace.

**Figure 3:**
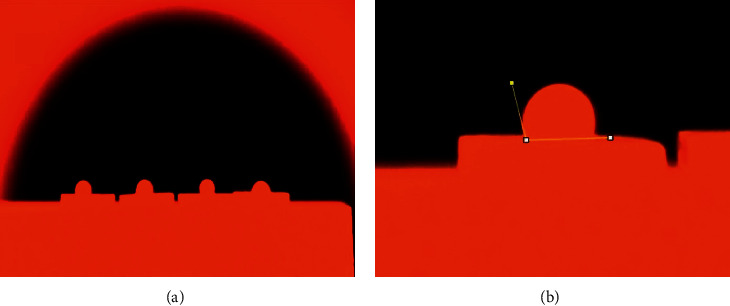
(a) Zirconia-veneering ceramic assemblies in the tube furnace; (b) contact angle measurement by drawing two tangent lines (yellow lines), one to the zirconia surface and another to the feldspathic ceramic droplet.

Half of the specimens in each group were randomly subdivided (*n* = 16) to be subjected to the hydrophilicity test and contact angle measurement. 0.05 g of feldspathic ceramic powder (CERABIEN™ ZR, *A*_2_ dentin, Kuraray Noritake Dental Inc., Japan; lot number 024899) was mixed with the manufacturer's liquid (in a 1 : 1 ratio) and placed on each of the zirconia block using a plastic mold (3.3 mm in diameter and 3 mm in height). Afterward, the specimens were placed in a special tube furnace (silicon carbide furnace, NovaMavad Corp, Tehran, Iran) at constant temperature of 1500°C up to 120 min with a glass opening which made it possible to take images of specimens during the firing process (Figures 2 and 3). Images were taken by a digital camera (Canon EOS 80D, Japan) at 0, 3, 5, 10, 15, 20, 30, 40, 50, 60, 75, 90, 105, and 120 min and were analyzed by an ImageJ software (Rasband, WS, ImageJ, US National Institutes of Health, Bethesda, Maryland, USA, 1997–2018).

It should be noted that regardless of the initial shape of the feldspathic ceramic on zirconia block, when feldspathic ceramic-zirconia assemblies undergo firing process, the feldspathic ceramic melts on the zirconia surface and starts to form a droplet. By the progression of firing process, the contact angle between the zirconia surface and droplet of feldspathic ceramic decreases. Thus, two tangent lines were drawn in each image taken: one to the zirconia surface and another to the feldspathic ceramic droplet. Then, the angle between the two lines was measured (Figure 3(b)) [37]. This process was repeated three times, and the average of three measurements was reported as contact angle.

### 2.4. Shear Bond Strength Measurement

**Figure 4:**
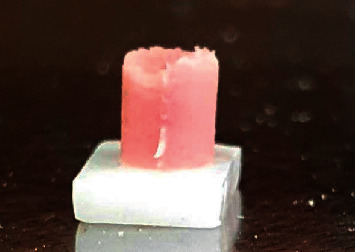
Zirconia-veneering ceramic assembly used for the shear bond strength test.

The remaining specimens in each group were used for shear bond strength evaluation. The feldspathic ceramic powder was mixed with the manufacturer´s liquid and condensed on each zirconia specimen using a standard mold with a diameter of 3.3 mm and height of 3 mm (Figure 4). As mentioned in the previous section, the zirconia blocks in the FC and Zr/FC groups received plasma-treated feldspathic ceramic powder, and the specimens in the control and Zr groups received untreated feldspathic ceramic powder. Finally, the zirconia-feldspathic ceramic assemblies were fired in a ceramic furnace according to the manufacturer's instructions. After firing, the specimens were cooled at room temperature for one day. After the cooling process, the specimens were subjected to thermocycling in water baths between 5°C and 55°C with a dwell time of 15 s for 3500 cycles. Then, the shear bond strength was measured using a universal testing machine (STM-20, Santam Co., Tehran, Iran) at a cross-head speed of 0.5 mm/min and load cell of 200 N. The load at failure in Newton was recorded, and the cross-sectional area of feldspathic ceramic bonded on the zirconia core at the site of fracture was measured in mm to calculate the bond strength in MPa (the fracture load divided by surface area). The mode of failure was evaluated under a stereomicroscope (SMZ800, Nikon Instruments Inc., New York, USA).

### 2.5. Statistical Analyses

Data of both contact angle and shear bond strength measurements were analyzed using one-way analysis of variance (ANOVA) with repeated measures test in IBM SPSS Statistics 25 (IBM SPSS Statistics for Windows, Version 25.0. Armonk, NY: IBM Corp.). The statistical significance was considered less than 0.05.

## 3. Results

**Figure 5:**
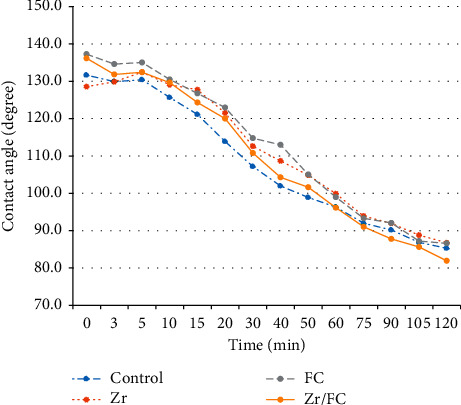
Correlation between the contact angle values and time in the four experimental groups. Control: specimens with no surface treatment, Zr: plasma exposure on zirconia, FC: plasma exposure on feldspathic ceramic, Zr/FC: plasma exposure on both zirconia and feldspathic ceramic.

The contact angle measurements (in degrees) of the experimental groups at different time intervals are summarized in Table 1. First, the contact angles at a specific firing time between different experimental groups were compared. The highest contact angle value was observed in Zr group at 120 min, followed by FC, C, and Zr/FC, groups, respectively. However, there was no statistically significant difference between the experimental groups at any of the time intervals (*P*=0.32). Then, the contact angles of samples over time (0 to 120 min) during firing in each experimental group were compared. The mean contact angle values in each group decreased significantly over time (*P* < 0.001).  Figure 5 shows the correlation between the contact angle and time in the four experimental groups.

**Figure 6:**
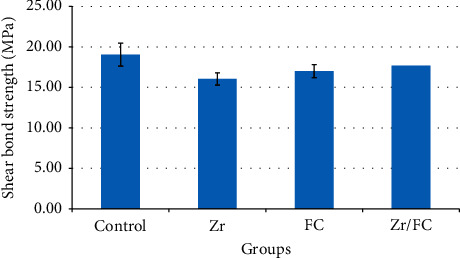
The mean shear bond strengths (MPa) in the four experimental groups. Control: specimens with no surface treatment, Zr: plasma exposure on zirconia, FC: plasma exposure on feldspathic ceramic, Zr/FC: plasma exposure on both zirconia and feldspathic ceramic.

Regarding the shear bond strength values, the highest value was observed in the control group followed by Zr/FC, Zr, and FC groups. However, there was no significant difference in the mean shear bond strength values between the control, Zr, FC, and Zr/FC groups (*P*=0.21).  Figure 6 shows the mean shear bond strength values for the four experimental groups.

**Figure 7:**
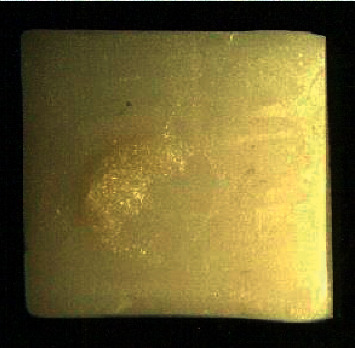
Mixed failure mode observed in zirconia-feldspathic ceramic assembly.

Furthermore, the results of the failure mode in the experimental groups are summarized in Table 2. The failure modes of the zirconia-feldspathic ceramic assemblies were predominantly mixed in all the experimental groups such that the fracture was at the interface and then through the feldspathic veneer which partly remained at the interface as shown in Figure 7.

## 4. Discussion

Despite increasing interest in yttria-stabilized zirconia-based all-ceramic restorations in recent years, debonding or chipping of veneering ceramic has been one of the most reported clinical failures associated with full ceramic restorations [38]. As a result, various zirconia surface treatment methods have been proposed and evaluated to improve the bond of veneering ceramic to zirconia core. Among these methods, the application of nonthermal plasma has gained researchers' attention in recent decade [39, 40]. This method was first introduced to improve the bond strength between resin cement and the zirconia core [24] and then was used to enhance zirconia-veneering ceramic bond. However, previous studies have mostly focused on applying nonthermal argon plasma on the zirconia surfaces [28, 41, 42]. By reviewing the literature, we found only one study in which methane (CH_4_), oxygen (O_2_), and argon plasma were used and their subsequent effects on the bond strength were investigated.

It has been reported that the helium plasma jet is more efficient than argon since the difference between the metastable state and ionization energy for helium plasma is 3.9 eV which is less than that of argon (4.3 eV) [33]. Moreover, the efficacy of helium plasma and argon plasma on the hydrophilic characteristics of polymethylmethacrylate (PMMA) has been investigated in a study conducted by Wang et al. [34]; the helium plasma was more effective in improving the hydrophilic properties of PMMA and also produced more active species compared to the argon plasma. Hence, we assumed that the helium might be more efficient in improving the hydrophilic properties of the zirconia surface which would result in a stronger bond between the zirconia and veneering ceramic, and this is why we chose helium plasma over argon in the present study.

Furthermore, the zirconia surface was the only component in zirconia-porcelain restorations that received plasma treatment in the previous studies. It has been suggested that the application of argon plasma reduces the organic matter and generates reactive species on the surface [31, 32]. Therefore, we assumed that applying plasma on the feldspathic veneering powder might have a similar effect, and plasma was applied on both the zirconia surface and feldspathic veneering ceramic powder in this study.

It is believed that an increase in the surface energy of zirconia might result in more favorable bonding properties [43]. Contact angle measurement is a general approach to evaluate the surface energy [44], and sessile drop method is the most commonly used method to measure the contact angle. In this method, a liquid droplet is placed on the surface, and the angle between the surface and the droplet is measured. Many studies have used this method to evaluate the surface energy and wettability before and after plasma application at room temperature [28, 29, 37]. However, it seems that the wettability of the zirconia surface by feldspathic slurry plays a more critical role in predicting the bond strength. Thus, we used a new method first employed by Marefati et al. [12] to evaluate the wettability of the zirconia surface by feldspathic ceramic slurry instead of a water droplet. We put feldspathic ceramic slurry on each zirconia sample. After placing the zirconia-feldspathic ceramic assemblies in the furnace, the contact angles were measured at different time intervals.

### 4.1. Contact Angle Measurement

The results showed that there was no statistically significant difference in the obtained contact angle values among the control, Zr, FC, and Zr/FC groups during the firing process at different time intervals from 0 to 120 min (*P*=0.32). These results were in contrast with those of Ji et al. [28] and Lee et al. [41] who reported that the application of argon nonthermal plasma on zirconia core for 10 min decreased the contact angle between water droplet and zirconia surface significantly. However, in this study, we applied the helium plasma on the samples for 60 s as suggested by Lopes et al. [37]. Different types of used plasma gas, as well as longer exposure time used in the previous studies, might contribute to different results of this study and the previously published studies. It is possible that longer exposure time is needed for helium plasma to affect the wettability of zirconia surface properly and decrease the contact angle.

It should be noted that although argon plasma can improve the surface wettability by producing reactive species on the surface, helium plasma might not have a similar effect on the surface, and thus further studies evaluating the surface characteristics of zirconia after nonthermal helium plasma exposure are required to verify this assumption.

Furthermore, the brand of zirconia might slightly affect the final contact angle. Lopes et al. [37] studied the effect of nonthermal argon plasma treatment on the contact angle of two brands of zirconia; according to their results, after the plasma exposure, Katana zirconia and Lava zirconia were significantly different from each other regarding the contact angle measurements. This is due to the fact that different brands could differ in surface qualities including surface polar groups, and this difference might result in different contact angles of two zirconia brands after the same surface treatment.

Our results also showed that the mean contact angle values in each group decreased significantly over time (*P* < 0.001), as this was expected because of the increased fusion of feldspathic ceramic to the zirconia core over time during the firing procedure in the furnace.

### 4.2. Shear Bond Strength Test

Regarding the shear bond strength, helium plasma could not significantly improve the bond strength of veneering ceramic to zirconia core. These results are in contrast with the results of the previous studies [18, 28, 29] that reported an improved bond between the veneering ceramic and zirconia after application of nonthermal argon plasma. According to Liu et al. [29] findings, different types of nonthermal plasma gases might have dissimilar effects on the zirconia-veneer bond; application of methane plasma and argon plasma significantly improved the bond strength of veneering ceramic to zirconia, while oxygen plasma adversely affected the bond, since it increased the porosities in the veneering ceramic adjacent to the veneer-zirconia interface and thus impaired the bonding. Nonthermal helium plasma might affect the veneering ceramic in the same manner as oxygen plasma; however, further studies employing interfacial porosity analysis are required to verify this speculation.

In a study done by Vilas Boas et al. [45], the shear bond strength between the zirconia and resin cement was evaluated. They used nonthermal argon plasma in one of their experimental groups. Accordingly, the plasma-treated group was excluded from the shear bond strength test due to the adhesive failure during the sample cutting procedure. It seems that in their study, the plasma application not only did not improve the bond strength but also led to the pretest failure in the samples. They concluded that plasma application had no significant effect on improving the bond strength.

In another study, Tabari et al. [24] evaluated the effect of different types of plasma including air, argon, oxygen, and a combination of argon and oxygen on the bond strength between zirconia and resin cement. They found out that only the air plasma and the combination of argon and oxygen plasma could increase the bond strength significantly. These results suggest that different plasma gases might have different effects on the bond strength values.

Another possible speculation is that helium plasma would potentially improve the bond strength if longer exposure time was used. In [27], the shear bond strength after 120 s of argon plasma application was significantly higher than that of 20 s exposure time. We used 60 s exposure according to the previous studies [32, 39, 40, 45]. It is possible that 60 s of exposure has not been sufficient for helium plasma to enhance the bond strength. As a result, future studies using nonthermal helium plasma with different exposure times would be beneficial for verifying the finding of the present study.

In the oral environment, dental restorations are subjected to thermal and mechanical fatigue and moisture as well. These factors could induce temporary deformation and defects at the material interfaces [46]. Thermocycling is a widely used method to simulate oral environment [43] which might affect the shear bond strength negatively [44]. According to Liu et al. [29], the shear bond strength between the zirconia and feldspathic ceramic decreased after thermocycling. In another study, the shear bond strength in the plasma-treated group was significantly higher than that of control group before thermocycling. However, after thermocycling, there was no significant difference in the shear bond strength values between the two groups [40]. In a study performed by Ahn et al. [39], the bond strength between the zirconia core and resin cement decreased more noticeably in the plasma-treated groups after thermocycling. This might have also happened in the present study such that thermocycling affected the bond strength values negatively, especially in the plasma-treated groups. In other words, it was possible that helium plasma had improved the bond strength in the plasma-treated groups, but after thermocycling, the plasma-treated groups experienced a drastic decrease in the bond strength values. It is believed that during thermocycling, the polar chemical groups are reorientated into the bulk of the material and decrease the surface energy [47]. In addition, the concentration of oxygen-containing functional groups and C/O atomic ratio reduce during aging process [48]. Therefore, thermocycling might have had a more prominent effect in decreasing the bond strength in the plasma-treated samples in this study. Another explanation is that yttria-stabilized zirconia stabilizes in its tetragonal phase after the sintering process, in which zirconia has greater mechanical strength and applicability in dental practice. The phase transformations in zirconia after sintering occur mainly when it is subjected to an aging process, such as in an aqueous environment or due to the temperature changes. Plasma application on zirconia was a process in which no heat emission occurred. However a transformation from the tetragonal phase of zirconia to monoclinic phase might have occurred after thermocycling, thus deteriorating the physical properties of zirconia surface and decreasing the bond strength. We did not compare the experimental groups before and after thermocycling, and this was a limitation of the present study.

Finally, shear bond strength has some limitations as the predominant mode of failure is usually cohesive within the substrate and not adhesive at the interface. This would indicate that the shear bond strength test cannot represent the actual interfacial bond strength values. However, this was not the case at the present study because the substrate was zirconia, a very high-strength ceramic. Therefore, no cohesive fracture within the zirconia substrate was observed, and the predominant failure was mixed in all the experimental groups such that the fracture was at the interface and then through the feldspathic veneer which partly remained at the interface (Figure 7). These findings are consistent with the results of previous studies [18, 28, 41].

It should be noted that the present study is the first study that has evaluated the effect of helium plasma on the contact angle and shear bond strength between the zirconia and feldspathic veneering ceramic. Further studies are required to assess the effect of helium plasma gas using longer plasma exposure times and also the effect of thermocycling on the bond strength between the plasma-treated zirconia and feldspathic veneering ceramic.

## 5. Conclusions

Within the limitations of the present study, it was concluded that 60 s of nonthermal helium plasma application applied on either zirconia core or feldspathic veneering ceramic powder had no significant effect on the wettability of the zirconia surface by feldspathic veneering ceramic and also on the shear bond strength between the zirconia core and feldspathic veneering ceramic.

## Figures and Tables

**Table 1:** Contact angle measurements (in degrees) of experimental groups at different time intervals in the furnace.

(min)	Control	Zr	FC	Zr/FC
0	131.66 ± 9.05	128.56 ± 12.56	137.25 ± 10.54	136.16 ± 11.38
3	129.90 ± 8.03	129.85 ± 10.55	134.63 ± 13.75	131.83 ± 9.15
5	130.39 ± 9.78	132.44 ± 9.94	135.04 ± 14.45	132.42 ± 10.57
10	125.65 ± 6.35	129.05 ± 10.39	130.50 ± 11.10	129.63 ± 10.16
15	121.12 ± 7.03	127.73 ± 7.36	126.73 ± 9.08	124.32 ± 8.50
20	113.88 ± 9.57	121.52 ± 7.34	122.98 ± 9.57	120.01 ± 9.05
30	107.18 ± 10.18	112.59 ± 8.41	114.74 ± 8.82	110.80 ± 10.33
40	101.99 ± 12.04	108.66 ± 9.89	112.97 ± 6.22	104.27 ± 11.19
50	98.90 ± 11.22	104.86 ± 9.63	105.04 ± 7.49	101.63 ± 11.31
60	96.31 ± 11.16	99.89 ± 8.48	98.95 ± 6.21	96.13 ± 11.78
75	92.00 ± 8.60	93.91 ± 6.41	93.30 ± 5.65	91.04 ± 9.59
90	90.16 ± 8.89	91.96 ± 7.59	92.08 ± 3.93	87.80 ± 5.33
105	86.88 ± 6.41	88.81 ± 6.69	87.19 ± 3.83	85.65 ± 6.41
120	85.28 ± 6.47	86.77 ± 3.86	86.59 ± 4.02	81.92 ± 7.85

Control: specimens with no surface treatment, Zr: plasma exposure on zirconia, FC: plasma exposure on feldspathic ceramic, Zr/FC: plasma exposure on both zirconia and feldspathic ceramic.

**Table 2:** The number of modes of failure observed in the experimental groups after shear bond test.

		Zr	FC	Zr/FC	Control
Failure mode	Adhesive	4	3	4	1
	Mixed	11	10	12	14
	Cohesive	1	3	0	1

Control: specimens with no surface treatment, Zr: plasma exposure on zirconia, FC: plasma exposure on feldspathic ceramic, Zr/FC: plasma exposure on both zirconia and feldspathic ceramic.

## Data Availability

The data that support the findings of this study are available from the corresponding author upon reasonable request.
